# Epithelial Cell Differentiation Regulated by MicroRNA-200a in Mammary Glands

**DOI:** 10.1371/journal.pone.0065127

**Published:** 2013-06-04

**Authors:** Kentaro Nagaoka, Haolin Zhang, Gen Watanabe, Kazuyoshi Taya

**Affiliations:** Laboratory of Veterinary Physiology, Department of Veterinary Medicine, Tokyo University of Agriculture and Technology, Tokyo, Japan; Wayne State University School of Medicine, United States of America

## Abstract

Mammary gland epithelial cells undergo periodic cycles of proliferation, differentiation, and involution. Many studies have reported that miRNAs, which are small, non-coding RNAs, influence a variety of biological processes during posttranscriptional regulation. Here, we found that one miRNA, miR-200a, was relatively highly expressed in epithelial cell-rich organs such as mammary glands, lung, and kidney in mice. In mammary glands, miR-200a expression increased during mid-pregnancy through lactation; its expression was stimulated by lactogenic hormone treatment of mammary epithelial cells. Lactogenic hormone also induced the expression of milk protein ß-casein mRNA (a marker of cell differentiation) and E-cadherin mRNA (a marker of epithelial cells). However, knockdown of miR-200a prevented increases in ß-casein and E-cadherin mRNA expression. Protein analysis revealed that E-cadherin signal was decreased and ZEB1 (a marker of EMT) was increased following miR-200a knockdown. Finally, in a three-dimensional culture system modeling lumen-containing mammary ducts, miR-200a knockdown decreased the cavity formation rate and suppressed claudin-3 and par-6b expression, indicating reduced epithelial cell polarity. These observations suggest that miR-200a is important for maintaining the epithelial cell phenotype, which contributes to lactogenic hormone induction of cellular differentiation in mammary glands.

## Introduction

The mammary gland is a unique and dynamic organ that exhibits distinct phases throughout the female reproductive cycle. These successive physiological stages are characterized by proliferation, differentiation, and apoptosis of the mammary epithelial tissue. Numerous hormones and growth factors exhibit positive or negative effects that tightly regulate these transitions [1], [2]. During mammary gland differentiation, lactogenic hormones such as prolactin and glucocorticoid induce mammary epithelial cells to undergo growth arrest and initiate milk protein production [3]. Since the lactating mammary glands synthesize larger quantities of proteins than other organs, precise gene regulation is important for coordinating cellular and tissue remodeling during differentiation stages.

In the past decades, mammalian gene regulation has become more complicated than the central dogma of molecular biology. Less than 2% of the mammalian genome contains protein-coding regions, and much larger non-coding RNAs (ncRNAs) are transcribed [4]. Although ncRNAs are grouped into several classes based on the transcript size, increasing evidence indicates that this group of RNAs is vast and varies in a similar manner to their protein coding mRNA counterparts. Generally, organisms that are more complex exhibit greater numbers of ncRNAs [5].

MicroRNAs, small ncRNAs comprised of 18–25 bases, are known to be involved in regulating various cellular processes [6]. They regulate gene expression at the posttranscriptional level by binding their target mRNAs through base-paring interactions to subsequently induce translational repression or mRNA degradation [7]. Since most miRNAs described thus far regulate crucial cell processes such as proliferation, differentiation, and apoptosis, many miRNAs have been implicated in mammary gland development and tumorigenesis [8].

In our previous study, we conducted miRNA microarray analysis during mammary epithelial cell differentiation in mice and found that miR-101a may regulate cell proliferation by targeting COX-2 expression, which may be important for the differentiation and involution of mammary glands [9]. Similar to miR-101a, increased miR-200a expression was observed in differentiated epithelial cells. Previously, Galio *et al* reported that miR-200 is expressed in luminal cells of mammary gland during the second half of pregnancy in sheep [10]. Many studies have highlighted the importance of miR-200a in tumor progression and metastasis and suggested that miR-200a plays a crucial role in maintaining epithelial cell phenotype by targeting transcriptional repressors of E-cadherin [11]–[13].

E-cadherin is a well-known component of the adherens junction involved in cell polarity. The mammary gland develops as a branching network of interconnecting tubular ducts that culminate in alveoli or terminal end buds (TEBs). TEBs become lumen when the interior-most cells undergo apoptosis in response to reproductive hormones [14], [15]. The remaining epithelial cells lining the ducts become polarized with apical (luminal) and basolateral surfaces. During pregnancy and lactation, the mammary glands undergo proliferation and differentiation into a fully branched ductal network that orchestrates a secreted duct system capable of producing milk protein [16]. Establishing cell polarity in mammary epithelial cells is also important to generate high yields of milk protein during mammary gland development [17], [18].

In the present study, to better understand the importance of miR-200a during mammary gland development, we confirmed the expression profile of miR-200a in both mouse mammary gland tissues and in mammary epithelial cells *in vitro*. We also demonstrated the role of miR-200a in epithelial cell differentiation and cell polarity through miR-200a-knockdown experiments.

## Materials and Methods

### Animals

ICR mice were purchased from SLC (Sizuoka, Japan) and maintained at 23°C±1°C under a 14-h lighting schedule (lights on from 07∶00 to 21∶00). Food and tap water were given *ad libitum*. All experiments with mice were performed according to the guidelines of the Institutional Animal Care and Use Committee of Tokyo University of Agriculture and Technology. The day of the observation of vaginal plug was designated day 0 of pregnancy. The day of parturition was designated as day 0 of lactation. Mammary glands were collected from the mice, frozen immediately, and stored at −70°C.

### Cell culture and transfection

Mouse mammary gland epithelial cells, EpH4, were routinely maintained in DMEM containing 5 µg/mL insulin, 10% fetal bovine serum (FBS), and antibiotic-antimycotic (Invitrogen, Carlsbad, CA). To induce differentiation, confluent cells were incubated for 3 days with daily replacement of DMEM containing 10% FBS and a lactogenic hormone mix (DIP), 1 µM dexamethasone, 5 µg/mL insulin, and 5 µg/mL prolactin (Sigma, St. Louis, MO).

Transfection of anti-miR-200a inhibitors (Invitrogen) was performed using Lipofectamine 2000 (Invitrogen) according to the manufacturer's instructions. At 24 h after transfection, cells were treated with cell differentiation medium.

For matrigel three-dimensional (3D) cultures, cells were trypsinized, resuspended in assay media, and plated as single cells on 100% Matrigel-coated glass coverslips in 24-well tissue culture plates. The assay media was composed of DMEM supplemented with 2% Matrigel, 5 µg/mL insulin, 1 µg/mL hydrocortisone, and 3 µg/mL prolactin, and was changed every 3 days. To visualize changes in the morphology of cavity structures, immunofluorescence analysis for zona occludens (ZO)-1 was conducted.

### Quantitative real-time PCR

Complementary DNA (cDNA) was synthesized using M-MLV reverse transcriptase (Invitrogen) according to the manufacturer's instructions. The amount of cDNA or miRNA targets was determined based on real-time PCR results. Oligonucleotide primers were selected using a web-based Primer3 software and are listed in Table S1. Primer sets for miRNA were purchased from Invitrogen. PCR reactions were run using SYBR Premix Ex Taq II (TaKaRa Bio), and the expression of each target mRNA or miRNA relative to ß-actin mRNA or 5S rRNA was determined using the 2^−ΔΔCT^ method.

### Western blot analysis

Whole-cell lysates were prepared in RIPA buffer (50 mM Tris-HCl, 150 mM NaCl, 1 mM EDTA, 1 mM NaVO_4_, 50 mM NaF, 0.1% SDS, 1% Triton-100, and Protease Inhibitor Cocktail). Protein samples (15 µg) were separated by electrophoresis on 10% SDS-PAGE gels and transferred onto nitrocellulose membranes (Immobilon; Millipore, Bedford, MA). Membranes were blocked with 5% skimmed milk at room temperature for 1 h before incubation with anti-E-cadherin (BD bioscience, Franklin Lakes, NJ), anti-ZO-1 (Invitrogen), anti-ZEB1, anti-ß-catenin (Cell Signaling, Beverly, MA) or anti-tubulin (Sigma) at 4°C for 12 h. After incubation, the membranes were washed 4 times in phosphate-buffered saline (PBS) containing Tween-20, incubated with horseradish peroxidase-conjugated anti-mouse or anti-rabbit IgG (GE healthcare UK Ltd., Buckinghamshire, UK) at room temperature for 1 h, and again washed 4 times in PBS-Tween 20. Protein bands were detected using the ECL Plus Western Blotting Detection System (GE Healthcare).

### Immunofluorescence

EpH4 cells were cultured on Matrigel-coated glass coverslips and fixed in 4% paraformaldehyde for 30 min. Cells were permeabilized using 0.5% Triton X-100/PBS for 10 min, blocked with 2% BSA/PBS for 1 h, treated with anti-E-cadherin (BD bioscience), anti-ZO-1 (Invitrogen), anti-ZEB1 (Cell signaling) or anti-Cld3 (Abcam, Cambridge, MA) for 1 h, and incubated with Alexa 488-conjugated anti-mouse IgG or Alexa 568-conjugated anti-rabbit IgG for 1 h. Slides were then mounted with ProLong Gold with DAPI (Invitrogen). Images were captured using an inverted confocal microscope (LSM710: Carl Zeiss, Oberkochen, Germany).

### Statistical analysis

The data are presented as mean ± SEM of 3 independent experiments, each performed in triplicate. The level of significance was analyzed using one-way analysis of variance, followed by multiple range tests (GraphPad Prism5). Differences were considered statistically significant when P<0.05.

## Results

### Expression of miR-200a in mice tissues

**Figure 1:**
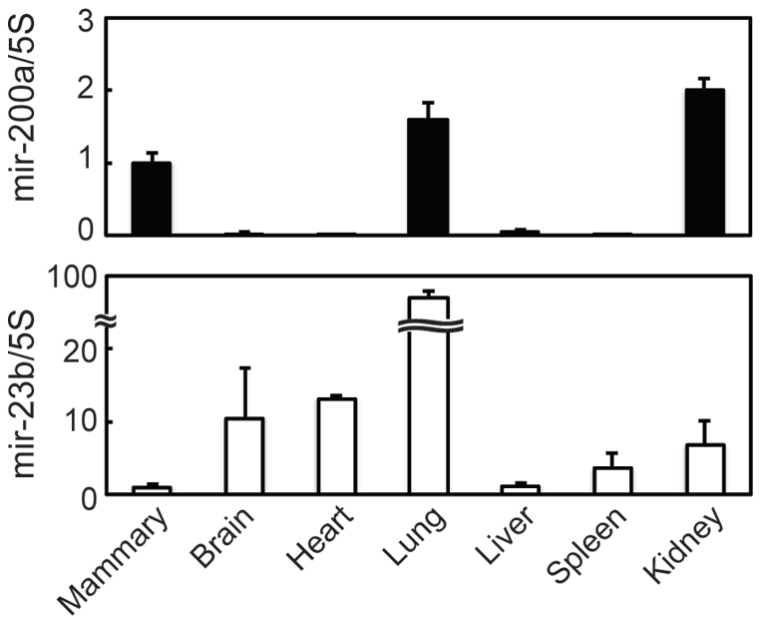
Tissue expression of miR-200a in mice. Mice brain, heart, lung, liver, spleen, kidney, and mammary glands were collected on day 7 of lactation (n = 3 animals), and expression of miR-23b and miR-200a were analyzed by real-time PCR.

To confirm the tissue expression of miR-200a and miR-23b (as a control) in mice, we conducted real-time PCR analysis using cDNAs from brain, heart, lung, liver, spleen, kidney, and mammary glands (Fig. 1). The results showed high expression of miR-200a in the lung, kidney, and mammary glands, which are organs composed of epithelial cells. miR-23b expression was observed in most tissues except in the liver and mammary glands.

### Changes in miR-200a expression during mammary epithelial cell differentiation

**Figure 2:**
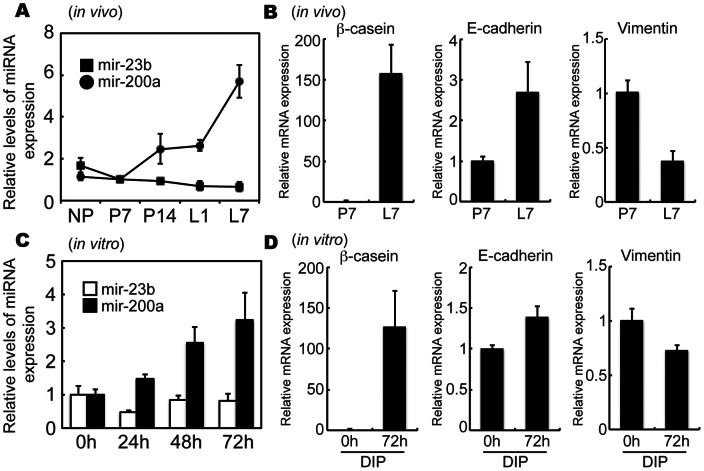
Changes in miR-200a, ß-casein, E-cadherin and vimentin mRNA expression during mammary epithelial cell differentiation. (A and B) Mammary glands were collected at non-pregnancy (NP), days 7 (P7) and day 14 (P14) of pregnancy, and days 1 (L1) and day 7 (L7) of lactation (n = 3 animals). (C and D) EpH4 cells were treated with DIP for 0, 24, 48, or 72 h. Expression of miR-200a, ß-casein, E-cadherin and vimentin mRNA expression were analyzed by real-time PCR. All experiments were repeated three times.

To define the involvement of miR-200a in mammary gland development, mammary glands were collected from non-pregnant mice, mice at days 7 and 14 of pregnancy, and mice at days 1 and 7 of lactation. Real-time PCR analyses showed that miR-200a expression gradually increased from mid-pregnancy (P14) to lactation (Fig. 2A). On the other hand, there was no change in miR-23b expression. Expression of ß-casein (a mammary gland differentiation marker) and E-cadherin (an epithelial cell marker) was increased during the lactation stage (Fig. 2B). A positive correlation was observed for the expression of miR-200a. In contrast, expression of vimentin, a mesenchymal marker, was decreased during the lactation stage.

To mimic *in vivo* states, EpH4 cells were induced to undergo lactogenic differentiation by DIP treatment for 72 h. Increased expression levels of miR-200a, but not miR-23b, was observed after 48 h and 72 h DIP treatment (Fig. 2C). ß-casein and E-cadherin expression was also increased after 72 h DIP treatment (Fig. 2D).

### Effect of miR-200a knockdown on EpH4 cell differentiation

**Figure 3:**
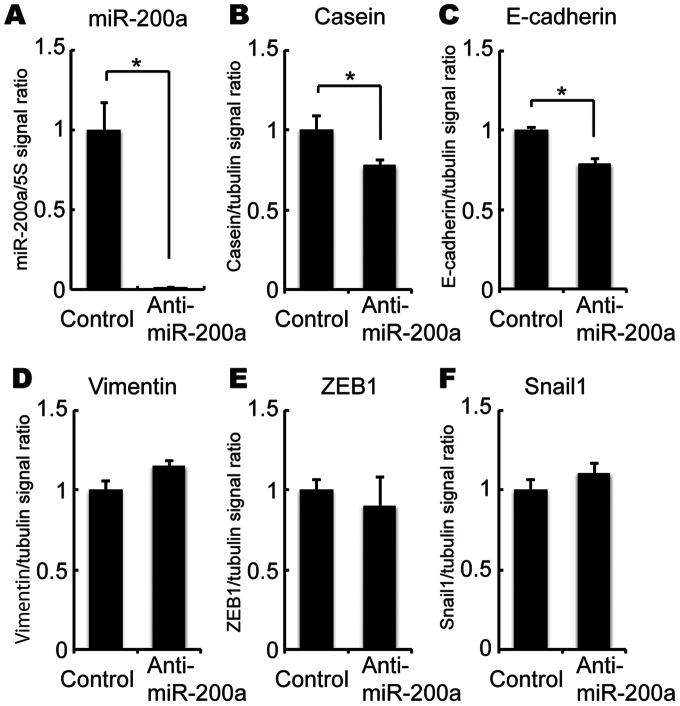
Effect of miR-200a knockdown on ß-casein, E-cadherin, vimentin, ZEB1 and Snail1 mRNA expression. EpH4 cells were transfected with anti-miR-200a or control antisense. Twenty-four hours after transfection, cells were treated with or without DIP for 72 h. After DIP treatment, expression of miR-200a (A), ß-casein (B), E-cadherin (C), vimentin (D), ZEB1 (E) and Snail1 (F) mRNA were analyzed by real-time PCR. All experiments were repeated three times.

To investigate whether miR-200a controls mammary gland epithelial cell differentiation, we conducted loss-of-function experiments. Before performing the DIP treatment, we transfected oligoribonucleotide anti-sense miR-200a into EpH4 cells. At 24 h after transfection, we began DIP treatment for 72 h; at this time point, we confirmed the knockdown of miR-200a by real-time PCR (Fig. 3A). As shown in Fig. 3B, the expression of ß-casein mRNA after DIP treatment was decreased in transfection with anti-miR-200a compared to transfection with the anti-sense RNA control. Similar to ß-casein, the miR-200a knockdown decreased E-cadherin mRNA levels under the DIP treatment (Fig. 3C). No significant differences were observed in the vimentin, ZEB1 and Snail1 (EMT markers) expression levels (Fig. 3D–F).

**Figure 4:**
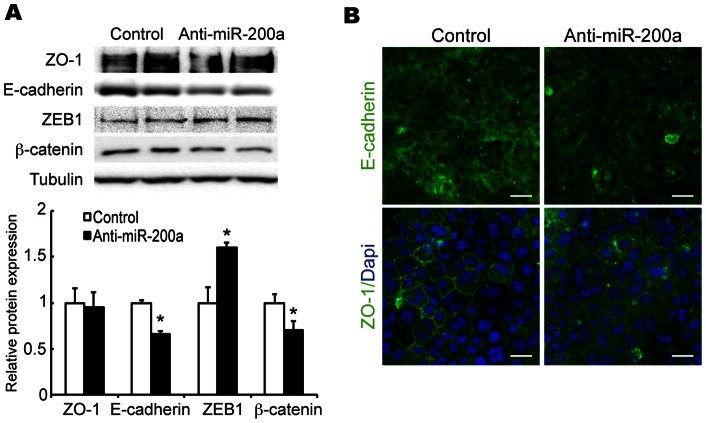
Effect of miR-200a knockdown on E-cadherin protein expression. (A) E-cadherin, ZO-1, ß-catenin and ZEB1 protein levels were determined by western blot analysis. (B) Confluent EpH4 cells transfected with control or anti-miR-200a antisense under DIP treatment were immunostained for E-cadherin and ZO-1. Scale bar  = 10 μm. All experiments were repeated three times.

**Figure 5:**
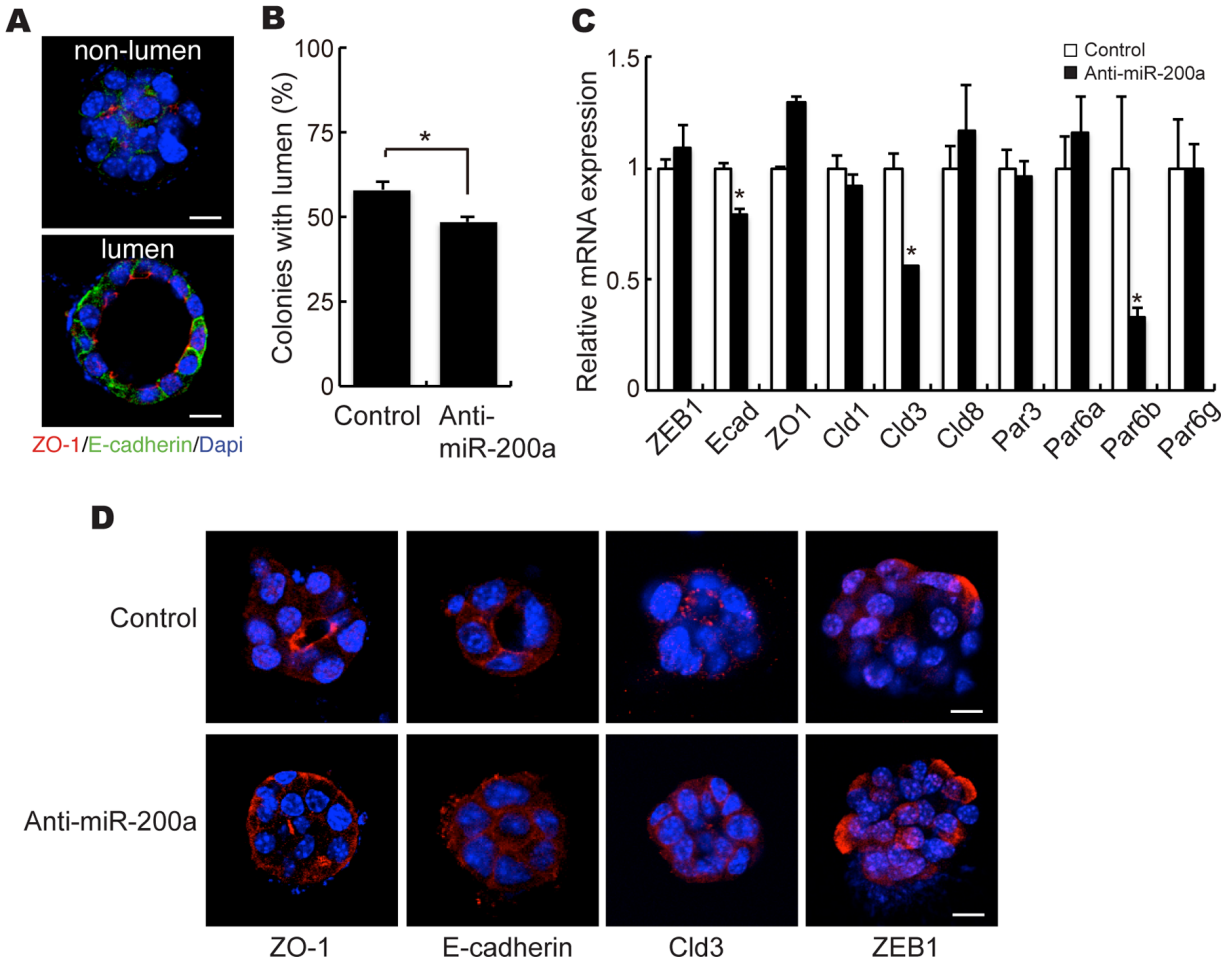
Effect of miR-200a knockdown on 3D cavity formation. (A) EpH4 cells grown in an anchorage-independent manner were immunostained for E-cadherin and ZO-1. In the confocal images of the colonies, the green color indicates immunostaining for E-cadherin, red color indicates immunostaining for ZO-1, and blue color indicates DAPI. Scale bar  = 10 μm. (B) Approximately 60% of EpH4 cells formed cavities with polarized cells when cultured in a suspension with matrigel. Knockdown of miR-200a resulted in a significant (p<0.05) reduction in the cavity formation (48%). (C) EpH4 cells (control vs miR-200a-knocked down) grew on matrigel, and expression of cell polarity-related genes was analyzed by real-time PCR. (D) EpH4 cells grew on matrigel were immunostained for ZO-1, E-cadherin, Claudin-3 and ZEB1. Scale bar  = 10 μm. All experiments were repeated three times.

Western blot analysis showed that ß-catenin (different marker of epithelial cell) as well as E-cadherin protein levels in DIP treatment were decreased by transfection with anti-miR-200a (Fig. 4A). Furthermore, results of the immunofluorescence analyses also showed that the E-cadherin signal decreased in anti-miR-200a-treated cells. A tight junction protein, ZO-1, did not change the protein level, but apical membrane localization was reduced and the signals were diffused throughout the cytoplasm (Fig. 4A and B). In contrast to these epithelial markers, ZEB1 protein (EMT marker) level was increased by the transfection with anti-miR-200a (Fig. 4A and Fig. 5D).

### Effect of miR-200a knockdown on 3D cavity formation

E-cadherin, ß-catenin and ZO-1 reduction led us to examine whether miR-200a is involved in cell polarity. EpH4 cell colonies grown in an anchorage-independent manner are polarized and contain central cavities; thus, they have been used as *in vitro* models to examine mammary duct formation (Fig. 5A) [19]. Cavity formation was reduced when miR-200a was knocked down (Fig. 5B). Real-time PCR analysis showed that the expression of cell polarity-related genes, claudin-3 and Par-6b, as well as E-cadherin mRNA expression, was decreased in miR-200a knockdown cells (Fig. 5C). In cavity formation, ZO-1 and claudin-3 were localized in apical regions, and E-cadherin was in lateral regions, respectively. However, these proteins were randomly distributed in the miR-200a knockdown colonies that did not form cavities (Fig. 5D).

## Discussion

It is well known that lactogenic hormones activate the Jak2-Stat5 cascade in mammary gland epithelial cells and alter the transcription of several mRNAs to inhibit cell proliferation and initiate cell differentiation and milk production [20]. Recently, miRNAs were described as fascinating new molecules that significantly control gene expression through posttranscriptional regulation. In the present study, we provide evidence suggesting that one miRNA, miR-200a, plays an important role in mammary gland epithelial cell differentiation during pregnancy and lactation. Several target genes of miR-200a have been identified by comparing normal and cancer epithelial cells, such as ZEB1, ZEB2, SIRT1, and KEAP1 [11]–[13], [21]. We did not confirm the exact target gene in this cell differentiation process, but ZEB1 is most likely because anti-miR-200a decreased ZEB1 protein level in mammary epithelial cells. More importantly, miR-200a expression was stimulated by lactogenic hormones, and miR-200a could control cell polarity and maintain epithelial phenotype by increasing E-cadherin, which has been implicated in controlling gene expression for milk production.

The miR-200 family consists of 5 members localized on 2 genomic clusters, miR-200a/b, miR-429 and miR-200c, miR-141 on chromosomes 1 and 12 in humans and on chromosomes 4 and 6 in mice [22]. We observed increased miR-141 as well as miR-200a expression during mammary epithelial cell differentiation; the expression was stimulated by lactogenic hormone (data not shown). miR-200a and miR-141 are thought to interact with the same target sites based on similarities in their seed sequences. These miRNAs are transcribed from different genomes, but a similar mechanism exists to transcribe miRNAs in response to lactogenic hormone. Recently, it was reported that c-myc inhibited miR-200b-200a-429 promoter activity and lactogenic hormone decreased c-myc expression in mammary epithelial cells [23], [24]. These observations suggest that decreasing c-myc level might be required for expression of miR-200a by lactogenic hormone. Further studies should be conducted to examine how lactogenic hormone controls miRNAs transcription and whether there is a functional difference between miR-141 and miR-200a.

The miR-200 family has been implicated in the epithelial-to-mesenchymal transition (EMT) that occurs during tumor invasion and metastasis [11], [25], [26]. EMT is also essential during embryogenesis, and a number of extracellular signals can convert epithelial cells into mesenchymal cells. EMT is associated with the loss of expression of proteins involved in the formation of cell-cell junctions, such as E-cadherin and ZO-1 [27]. The miR-200 family negatively regulates 2 transcription factors, ZEB1 and ZEB2, which have been characterized as transcriptional repressors of E-cadherin [28]. Overexpression of ZEB1 and ZEB2 decreases the levels of E-cadherin and makes cells adopt a mesenchymal phenotype that is better equipped to invade tissues and metastasize. Conversely, the down-regulation of ZEB1 and ZEB2 increase E-cadherin levels, and an epithelial phenotype is maintained. Stable expression of E-cadherin beginning in late-pregnancy through lactation is essential in the function of differentiated alveolar epithelial cells [29]. During mammary epithelial differentiation, lactogenic hormone-induced miR-200a maintains E-cadherin expression and enhances milk protein gene expression. Moreover, it is likely that E-cadherin maintenance occurs is due to the down-regulation of ZEB1 and ZEB2 by miR-200a.

Cell polarity is a critical feature of mammary gland epithelium *in vivo*, and thus, it has become an important parameter for evaluating experimental epithelial model *in vitro*. Loss of epithelial cell polarity has been correlated with increased cell proliferation [30]. The 3D culture system has been used as an *in vitro* model to examine mammary duct formation, in contrast to the conventional 2D monolayer culture system [31]. In this study, miR-200a knockdown reduced the number of colonies containing cavities and miR-200a-knocked down cells expressed a low level of claudin-3 and Par-6b mRNA compared to control cells. The claudin family of proteins constructs extracellular structures such as tight junctions, and the composition of claudins determines the network pattern and paracellular permeability of the tight junction of epithelial cells in each tissue [32]. In the mouse mammary gland, claudin-3 changes the gene expression levels throughout mammary gland development and is the dominant component of the tight junction in lactating mammary epithelial cells [33], [34]. Par-6b is one of the Par-6 homologs found in mice, and the Par-3/Par-6/aPKC complex localizes to the tight junction, is required for tight junction assembly, and establishes apical-basal polarity in various epithelial cells [35]–[38]. Moreover, Par-6b is essential for normal blastocyst formation in the mouse embryo [39]. Par-6b knockdown resulted in cavitation failure without compromising blastomere cleavage or compaction. Cavitation failure likely resulted from abnormal epithelial junctions [39]. It is unclear whether miR-200a directly regulates the expression of claudin-3 and Par-6b by binding to their mRNAs; however, the regulation of cell polarity genes as well as E-cadherin is important for mammary epithelial differentiation during pregnancy and lactation.

In conclusion, we showed that lactogenic hormone stimulated miR-200a expression during mammary epithelial differentiation and that miR-200a controlled E-cadherin and other cell polarity genes to maintain the epithelial cell phenotype. Many studies have attempted to identify the exact target gene of each miRNA and/or the mechanism of miRNA transcribed from the genome; however, these issues remain unclear. Further studies are necessary to address these questions using mammary glands since these organs were recently developed evolutionarily, making them good candidates to examining miRNA machinery.

## Supporting Information

Table S1 **Nucleotide sequences of the primers used for real-time PCR.**
(DOCX)
