# Supplementary material for: Epithelial Cell Differentiation Regulated by MicroRNA-200a in Mammary Glands
Source: PLoS One. 2013 Jun 4;8(6):e65127. doi: 10.1371/journal.pone.0065127 (PMC3672172; doi:10.1371/journal.pone.0065127)
Supplement: Table S1 — Nucleotide sequences of the primers used for real-time PCR. (DOCX) [file pone.0065127.s001.docx]

Table S1. Nucleotide sequences of the primers used for real-time PCR

| Gene | Forward | Reverse |
| --- | --- | --- |
| E-cadherin | 5′-CAA GGA CAG CCT TCT TTT CG-3′ | 5′-TGG ACT TCA GCG TCA CTT TG-3′ |
| Vimentin | 5′-ATG CTT CTC TGG CAC GTC TT-3′ | 5′-AGC CAC GCT TTC ATA CTG CT-3′ |
| β-casein | 5′-TTG AAC TGA CTG AAA CTG GA-3′ | 5′-GTC GAA TTC AAA TGA ATG CC-3′ |
| ZEB1 | 5′-GGG GCA TCT CAC ACT TTT GT-3′ | 5′-AAC GGC TGT GAA CCA AAA AC-3′ |
| Snail1 | 5′-AAA CCC ACT CGG ATG TGA AG-3′ | 5′-GAA GGA GTC CTG GCA GTG AG-3′ |
| ZO1 | 5′-GGG AGG GTC AAA TGA AGA CA-3′ | 5′-CAC AGC GAA ATG ATG GAA GA-3′ |
| Cld1 | 5′-TTT TCC CGA TGA CCT TTC TG-3′ | 5′-AGT TTG CAG GAT CTG GGA TG-3′ |
| Cld3 | 5′-GCA CCC ACC AAG ATC CTC TA-3′ | 5′-AGC CTG TCT GTC CTC TTC CA-3′ |
| Cld8 | 5′-TCC CAA GGC GTA CAG ATT TC-3′ | 5′-CAC TCT CCA CTG AGG CAT GA-3′ |
| Par3 | 5′-CAG ACT CAA GGC AGG AGA CC-3′ | 5′-GGG TGT GAG AAC AAC GTC CT-3′ |
| Par6a | 5′-TGA CAG TGA CGA TGA CAG CA-3′ | 5′-AGA GGC TGA ATC CGC TAA CA-3′ |
| Par6b | 5′-GTT GTC GTC ACC CTG GAA CT-3′ | 5′-AAC CTG GGG GCA TTT TTA TC-3′ |
| Par6g | 5′-GAT TAC AAC GCC CTG CAT CT-3′ | 5′-AGG AAA CAC GGA TGG AAC AG-3′ |
| β-actin | 5′-AGC CAT GTA CGT AGC CAT CC-3′ | 5′-CTC TCA GCT GTG GTG GTG AA-3′ |
